# Neutrophil extracellular trap interfering therapy: a potential therapeutic option for cancers?

**DOI:** 10.1097/JS9.0000000000000534

**Published:** 2023-06-07

**Authors:** Dhivyadharshini Gajendran, Vishnu Priya Veeraraghavan, Krishna Mohan Surapaneni, Lavina Prashar, Ullas Mony

**Affiliations:** a Centre of Molecular Medicine and Diagnostics (COMManD), Saveetha Dental College and Hospitals, Saveetha Institute of Medical and Technical Sciences, Saveetha University; b Departments of Biochemistry, Medical Education, Molecular Virology, Research, Clinical Skills and Simulation, Panimalar Medical College Hospital & Research Institute; c SMAART Population Health Informatics Intervention Center, Foundation of Healthcare Technologies Society, Panimalar Medical College Hospital & Research Institute, Varadharajapuram, Chennai, Tamil Nadu, India; d Department of Physiological Sciences, School of Medicine, Unza Ridgeway Campus, University of Zambia, Lusaka, Zambia

*Dear Editor*,

Neutrophils are the most abundant type of leucocytes (white blood cells), which play an important role in the innate immune response by providing defence against a number of bacterial and other microbial infections^1,2^. Immune cells, mostly neutrophils and epithelial cells form the cellular components of saliva. Since neutrophils rapidly react to the changes in the niche, they play a significant role in oral cancer immunology. A novel mechanism of neutrophil-mediated host response called NETosis was reported in 2004^3^. Neutrophils form neutrophil extracellular trap (NET) that protect the host from an array of infectious diseases have been reported to play a role in malignancy as well. This NET-mediated trapping mechanism of circulating tumour cells has been detected in a number of diverse types of cancers, including head and neck cancers. It has been reported that the tumour infiltrating neutrophils produces NETs and also lead to tumour progression and subsequently promote metastasis in many cancers including head and neck cancer^4^. In addition, they are known to contribute to the progression as well as regression of tumours in many oral cancers through multiple molecular mechanisms, such as the suppression of cytotoxic and helper T-cell responses. Also, these NETs are known to induce tumour angiogenesis contributing to the metastatic risk of tumours. Neutrophilia and a high neutrophil-to-lymphocyte ratio have been linked with poor prognosis in several head and neck cancers^5^. These changes may be due to the spread of the tumour to the lymph nodes. Thus, understanding their molecular basis would help in figuring out the tumour immune escape mechanisms, as well as their diagnostics and therapy.

How NETosis promotes tumour progression in cancers?: NETs in cancer were first identified by Demers *et al*.^6^ in a mouse model of chronic myelogenous leukaemia. The role of NETs in tumours varies according to the status of the immune system. On one hand, NETs induce apoptosis in head and neck squamous cell carcinomas^7^. On the other hand, NETs have been found to enhance the proliferation of tumours by elevating the expression of activation markers and also by inhibiting the apoptosis of tumour cells^8^. NETs also play a role in the protection of tumour cells from cytotoxicity by the suppression of CD8 + and natural killer (NK) cells in the tumour niche or microenvironment, thus promoting tumour survival and growth^9^. Analysing and understanding the molecular mechanisms underlying NET formation has revealed that there are some key molecules, receptors, and signalling pathways, such as the upregulation of cytokines and chemokines, impairment of immune cell cytotoxicity, and induction of angiogenesis during metastasis which also participate in both NETosis and the biological behaviour of tumours.

Significance of NETosis in cancer therapy: NETosis can be considered a double-edged sword. Circulating levels of NET components, such as cell-free DNA (cfDNA), neutrophil elastase (NE)–DNA, myeloperoxidase (MPO)–DNA, and citrullinated histone H3 (CitH3) can serve as beneficial biomarkers for several kinds of cancers. Blocking the formation of NETs via small-molecule drugs against DNase I, NE inhibitors, MPO inhibitors, and PAD4 inhibitors might also have great potential, and studies have revealed that targeting NETs has resulted in a significant reduction of tumour proliferation and metastasis as well as decreased resistance to radiotherapy, chemotherapy, and immunotherapy^10^. NET inhibitors can also be used in conjunction with immunotherapies or as adjuvants to other cancer drugs to enhance their effectiveness^9^. In addition, platelets developing from NETosis might protect circulating tumour cells from immune cells through their trapping properties. Metastasis can be minimized or stopped by depleting or blocking platelets by disrupting this interaction of platelets, tumour cells, and NETs. Hence, NETs might serve as an effective target for cancer treatment^11,12^. Observing the role of NETs in upgrading the metastatic potential of tumour cells, inhibiting their formation, or reducing their activity in cancers could be advantageous for better prognosis in the treatment of cancer. However, only limited studies are currently available, and the ongoing clinical trials also have not yet provided any clarification or clear inferences to target NETs. Currently, the challenge in targeting NETs is a lack of precise biomarkers that can predict an adequate response to NET-interfering therapy in patients. Further studies may reveal detailed information about the connections between NETosis, cancers, and other diseases and help in identifying appropriate strategies to effectively modulate the dysregulated NETosis and associated mechanisms.

## Ethical approval

Not applicable.

## Sources of funding

Not funded by any agency and received funding in any manner/form.

## Author contribution

D.G., V.P.V., K.M.S., L.P., and U.M.: conceptualization, data curation, and writing – original draft preparation; K.M.S.: conceptualization, study design, data curation, writing – original draft preparation, reviewing and editing, and visualization and supervision.

## Conflicts of interest disclosure

The authors declare no conflicts of interest.

## Guarantor

Dr Krishna Mohan Surapaneni.

## Data availability statement

This correspondence is based exclusively on resources that are publicly available on the internet and duly cited in the ‘References’ section. No primary data were generated and reported in this manuscript. Therefore, data have not become available to any academic repository.
